# Clinical significance of NUCB2 mRNA expression in prostate cancer

**DOI:** 10.1186/1756-9966-32-56

**Published:** 2013-08-16

**Authors:** Hongtuan Zhang, Can Qi, Liang Li, Fei Luo, Yong Xu

**Affiliations:** 1 National Key Clinical Specialty of Urology, Tianjin Key Institute of Urology, Second Affiliated Hospital of Tianjin Medical University, Tianjin, China; 2 Laboratory of Population and Quantitative Genetics, School of Life Sciences, Tianjin Medical University, Tianjin, China

## Abstract

**Background:**

Nucleobindin 2 (NUCB2) abnormal expression has been reported in gastric cancer and breast cancer. However, the role of NUCB2 in prostate cancer (PCa) remains unclear. The aim of the present study was to investigate the NUCB2 expression in PCa tissues and adjacent non-cancerous tissues and its potential relevance to clinicopathological variables and prognosis.

**Methods:**

NUCB2 mRNA expression was determined by real-time quantitative real time reverse transcriptase polymerase chain reaction in 180 pairs of fresh frozen PCa tissues and corresponding non-cancerous tissues. Kaplan-Meier analysis and Cox proportional hazards regression models were used to investigate the correlation between NUCB2 expression and prognosis of PCa patients.

**Results:**

Our results showed that the expression level of NUCB2 mRNA in PCa tissues was significantly higher than those in non-cancerous tissues. Our results indicated that the high expression of NUCB2 in PCa was associated with lymph node metastasis, preoperative PSA, Gleason score, and angiolymphatic invasion. Kaplan–Meier survival analysis showed that patients with high NUCB2 expression have shorter biochemical recurrence (BCR)-free survival time compared to patients with low NUCB2 expression. Multivariate analysis revealed that NUCB2 expression was an independent predictor of BCR-free survival.

**Conclusions:**

NUCB2 might play a positive role in PCa development and could serve as an independent predictor of BCR-free survival.

## Background

Prostate cancer (PCa) is one of the most frequently diagnosed malignancies and a common cause of cancer mortality in men in the Western hemisphere [1], which has become a major public health challenge. In China, the incidence of PCa has been increasing continually in the most recent years. Although we have made considerable advances in diagnosis and adjuvant therapy of PCa, the overall survival rate of PCa patients has not been improved markedly. The mechanism of its carcinogenesis, like other cancers, is still not fully understood. It is a clinically heterogeneous, multifocal disease. Carcinogenesis and mechanisms influencing progression and prognosis of PCa are a multi-step process, involving both genetic insults to epithelial cells and changes in epithelial-stromal interactions [2]. Conventional prognostic factors such as Gleason score, preoperative PSA levels or ratio of involved biopsies only insufficiently predict patient outcome for currently available therapies. They are even more limited in identifying insignificant PCa. Therefore, there is an urgent need for better understanding of PCa pathogenesis which may lead to more effective treatment strategies [3-5].

Nucleobindin 2 (NUCB2) has a characteristic constitution of functional domains, such as a signal peptide, a Leu/Ile rich region, two Ca^2+^ binding EF-hand domains separated by an acidic amino acid-rich region, and a leucine zipper [6,7], and has a wide variety of basic cellular functions [8-10]. NUCB2 is known to mainly express in key hypothalamic nuclei with proven roles in energy homeostasis [8]. Moreover, recent studies have indicated that NUCB2 is also expressed in various human peripheral tissues, including the stomach, pancreas, reproductive organs, and adipose tissues, with relevant metabolic functions, suggesting that NUCB2 signaling might participate in adaptative responses and in the control of body functions gated by the state of energy reserves [11]. NUCB2 has been studied in breast cancer and gastric cancer [12,13]. To the best of our knowledge, NUCB2 has not yet been studied in PCa. Little is known about the expression of NUCB2 in PCa, and data on its potential prognostic value in PCa are completely lacking. Therefore, we examined NUCB2 in PCa using quantitative real time reverse transcriptase polymerase chain reaction (qRT-PCR) to explore its clinical significance.

In this study, the mRNA expression of NUCB2 was measured in PCa tissues and adjacent non-cancerous tissues by qRT-PCR. We studied the correlation between the relative expression of NUCB2 and clinicopathological parameters to evaluate its clinical significance. Additionally, we assessed the influence of NUCB2 expression on the biochemical recurrence (BCR) of PCa patients.

## Materials and methods

### Patient and tissue samples

The study was approved by the research ethics committee of Tianjin medical university. Informed consent was obtained from all of the patients. All specimens were handled and made anonymous according to the ethical and legal standards. PCa samples (n = 180) and adjacent non-cancerous tissues (n = 180) were collected from patients with PCa who underwent radical prostatectomy and were diagnosed at the second hospital of Tianjin medical university between 1999 and 2010 were retrieved for the study. None of the patients received androgen deprivation treatment, chemotherapy, or radiation therapy prior to radical prostatectomy. The tissue samples were snap-frozen in liquid nitrogen and stored at −80°C until used. The histopathology of each specimen was reviewed on the HE-stained tissue section to confirm diagnosis and tumor content at least 70% of tumor cells in the tissue sample. The following biochemical and pathological parameters were recorded: biochemical relapse, preoperative serum prostate-specific antigen (PSA), Gleason score, pathological stage, lymph node status, angiolymphatic invasion, margin status, and seminal vesicle invasion. The time to biochemical relapse was defined as the period between surgical treatment and the measurement of two successive values of serum PSA level ≥ 0.2 ng/ml.

### Isolation of RNA and qRT-PCR analysis

qRT-PCR was performed to determine the expression of NUCB2 mRNA. Briefly, the total RNA was extracted from frozen tissue by homogenization with a power homogenizer in TRIzol Reagent (Applied Invitrogen, Carlsbad, CA, USA) according to the manufacturer’s protocol (Life Technologies) and reverse-transcribed to generate cDNA (PrimeScript RT–PCR kit; Takara Bio). Human β-actin was amplified as an endogenous control. The levels of mRNA encoding were quantified by real-time PCR with the Applied Biosystems 7900HT Fast Real-Time PCR System using SYBR Premix Ex Taq (Applied Takara Bio). The sequences of the primers were as follows: human NUCB2 forward 5-AAAGAAGAGCTACAACGTCA-3′ and reverse 5′-GTGGCTCAAACTTCAATTC-3′; human β-actin forward 5′-TGACGTGGACATCCGCAAAG-3′ and reverse 5′-CTGGAAGGTGGACAGCGAGG-3. The PCR conditions included an initial denaturation step of 94°C for 2 min, followed by 35 cycles of 94°C for 30 s, 60°C for 20 s, 72°C for 2 min, and a final elongation step of 72°C for 10 min. All qRT-PCRs were performed in triplicate. The relative gene expression was calculated by the equation 2^-ΔΔCT^.

### Statistical analysis

qRT-PCR data were calculated with StepOne Software v2.1 (Applied Biosystems, Carlsbad, CA). Measurement data were analyzed by Student’s t-test, while categorical data were analyzed by chi-square test. The postoperative survival rate was analyzed with Kaplan–Meier method, and the log-rank test was used to assess the significance of differences between survival curves. The statistical analyses were performed using SPSS 16.0 software (SPSS, Chicago, IL, USA). All differences were considered statistically significant if the P value was <0.05.

## Results

### NUCB2 mRNA expression in PCa and adjacent non-cancerous tissues

The expression of NUCB2 mRNA was detected and analyzed in 180 pairs of PCa and adjacent non-cancerous tissues. The qRT-PCR results showed that the NUCB2 mRNA level was significantly higher in PCa tissues compared to that in adjacent non-cancerous tissues.

### Relationship between NUCB2 mRNA expression and clinicopathological variables

The mRNA expression of the NUCB2 was categorized as low or high in relation to the median value. We investigated the relationship between NUCB2 mRNA expression status and commonly used clinicopathological parameters in PCa. The association of NUCB2 mRNA expression with the clinicopathological parameters of PCa patients is shown in Table 1. The upregulation of NUCB2 mRNA in PCa tissues was correlated with the higher Gleason score (P < 0.001), the higher level of preoperative PSA (P = 0.004), the positive lymph node metastasis (P = 0.022), and the positive angiolymphatic invasion (P = 0.004). However, the NUCB2 mRNA expression was not related to age, seminal vesicle invasion, pathological stage, and surgical margin status.

**Table 1:** Main characteristics of studies included in this meta-analysis

		**NUCB2 mRNA expression**			
**Variable**	**Group**	**High**	**Low**	**Total**	**P value**
Age					0.100
	<70	43 (44.3%)	54 (55.7%)	97	
	≥70	47 (56.6%)	36 (43.4%)	83	
Lymph node metastasis					0.022
	Negtive	77 (47.2%)	86 (52.8%)	163	
	Positive	13 (76.5%)	4 (23.5%)	17	
Surgical margin status					0.578
	Negtive	82 (49.4%)	84 (50.6%)	166	
	Positive	8 (57.1%)	6 (42.9%)	14	
Seminal vesicle invasion					0.202
	Negtive	67 (46.2%)	78 (53.8%)	145	
	Positive	23 (65.7%)	12 (34.3%)	35	
Clinical stage					0.880
	T1	52 (50.5%)	51 (49.5%)	103	
	T2/T3	38 (49.4%)	39 (50.6%)	77	
Preoperative PSA					0.004
	<4	1 (20%)	4 (80%)	5	
	4-10	23 (35.9)	41 (64.1%)	64	
	>10	66 (59.5%)	45 (40.5%)	111	
Gleason score					
	<7	35 (35.4%)	64 (64.6%)	99	<0.001
	7	19 (55.9%)	15 (44.1%)	34	
	>7	36 (76.6%)	11 (23.4%)	47	
Angiolymphatic invasion					0.004
	Negtive	66 (44.9%)	81 (55.1%)	147	
	Positive	24 (72.7%)	9 (27.3%)	33	

### NUCB2 mRNA expression to predict clinical outcome after radical prostatectomy

**Figure 1:**
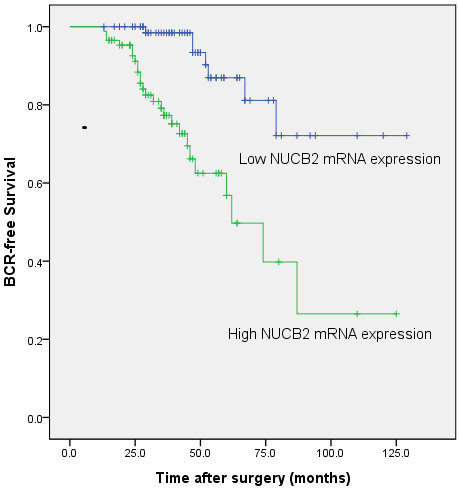
**Associations between NUCB2 expression and BCR-free time after radical prostatectomy in PCa patients.** Patients with high NUCB2 expression showed significantly shorter BCR-free survival than those with low NUCB2 expression (P < 0.001, log-rank test).

To examine if NUCB2 expression level is a significant predictor of BCR-free time after radical prostatectomy, Kaplan-Meier curves were plotted between high or low NUCB2 mRNA and BCR-free time. The low NUCB2 mRNA expression had significantly longer BCR-free time after radical prostatectomy compared to patients with high NUCB2 mRNA expression (P < 0.001; Figure 1). In univariate analysis with Cox proportional hazards model, Gleason score, NUCB2 expression, and seminal vesicle invasion were confirmed as significant prognostic factors for BCR-free survival times whereas age, angiolymphatic invasion, surgical margin status, pathological stage and preoperative PSA were not significant factors (Table 2). Furthermore, the multivariate analyses showed that the upregulation of NUCB2 mRNA, higher Gleason score, and Seminal vesicle invasion were independent predictors of shorter BCR-free survival (Table 2).

**Table 2:** Prognostic value of NUCB2 mRNA expression for the biochemical recurrence-free survival in univariate and multivariate analyses by Cox regression

	**Univariate analysis**			**Multivariate analysis**		
**Covariant**	**Exp (B)**	**95% CI**	**P value**	**Exp (B)**	**95% CI**	**P value**
NUCB2 expression	3.120	1.692–5.754	<0.001	2.900	1.569–5.360	0.001
Gleason score	1.703	1.280–2.265	<0.001	1.663	1.250–2.211	<0.001
Preoperative PSA	1.241	0.705–2.188	0.454			
Age	1.068	0.804–1.419	0.650			
Angiolymphatic invasion	1.084	0.814–1.443	0.580			
Surgical margin status	1.017	0.709–1.459	0.925			
PCa Stage	1.090	0.921–1.291	0.316			
Lymph node metastasis	1.140	0.850–1.528	0.381			
Seminal vesicle invasion	1.505	1.132–2.003	0.005	1.410	1.060–1.876	0.018

## Discussion

The identification of prognostic and predictive markers is clinically important, because PCa is heterogenous in respect to genetics, and variable in biological and clinical features. PCa is a heterogeneous–multifocal disease with a clinical outcome difficult to predict [14,15]. It is of great significance to identify novel diagnostic and prognostic markers to understand this multifaceted disease process [16-19]. An accurate and early diagnosis is essential for efficient management of PCa [20]. Therefore, to complement improvements in the clinical management, substantial progress in the diagnostic pathway of PCa is urgently needed [21-23].

To our knowledge, this is the first report to investigate the association between NUCB2 and PCa. The main findings of the present study are as following three points. First, qRT-PCR analysis found that NUCB2 mRNA expression was upregulated in PCa tissues compared with those in adjacent non-cancerous tissues. Second, this is the first report to describe the significance of NUCB2 to preoperative PSA, gleason score, angiolymphatic invasion, lymph node metastasis of PCa patients. Third, we proved that NUCB2 expression was significantly associated with BCR-free survival of PCa patients. In support of this, Kaplan–Meier analysis of BCR-free survival showed that patients whose tumors had high NUCB2 expression tend to have a significantly shorter BCR-free survival, indicating that high NUCB2 level is a marker of poor prognosis for BCR-free survival of PCa patients. The multivariate analyses showed that the upregulation of NUCB2 was an independent predictor of shorter BCR-free survival in PCa patients. These results suggest that NUCB2 may play important roles in the pathogenesis and aggressiveness of PCa, and NUCB2 upregulation especially be associated with the unfavorable prognosis in PCa. The precise molecular mechanisms behind the altered expression of NUCB2 in PCa are unclear. Additional studies to investigate the real molecular mechanisms of altered expression of NUCB2 in the development or progression of PCa are essential.

Currently, the advantages of serum PSA as a general PCa biomarker are viewed with intense skepticism [24]. The present study shows that NUCB2 classical mRNA transcript expression levels, assayed by a specific qPCR in prostate tissue samples, can improve PCa management by making available important and independent differential prognostic information. A variety of algorithms and nomograms that calculate the probabilities of BCR-free survival after treatment have been used in order to direct clinicians into the most suitable treatment options for PCa patients [25]; nonetheless patients still present unforeseen disease course patterns. Cox proportional hazards model showed that high NUCB2 expression was an independent prognostic predictor for PCa patients. Therefore, NUCB2 could constitute a molecular prognostic marker for PCa patients, identifying who are more likely to have higher risk of BCR and need receive a more aggressive treatment. Our findings could help establish a more personalized medicine-focused approach, where not only PSA, but also other novel and promising biomolecules will contribute to the multifactorial repertoire of individualized PCa care.

## Conclusions

In conclusion, our data offer the convincing evidence for the first time that that NUCB2 mRNA were upregulated in PCa tissues. Our study revealed that NUCB2 is an independent prognostic factor for BCR-free survival in patients with PCa. High expression of NUCB2 in PCa is strongly correlated with preoperative PSA, gleason score, angiolymphatic invasion, and lymph node metastasis. These findings suggest that NUCB2 is a cancer-related gene associated with the aggressive progression and a BCR-free survival predictor of PCa patients. However, these results, which are based on a Chinese cohort, should be further confirmed in other populations of patients with PCa. Our findings suggest that NUCB2 might be used as a new biomarker and a potential therapeutic target for PCa.

## Consent

Written informed consent was obtained from the patient for publication of this report and any accompanying images.

## Abbreviations

NUCB2: Nucleobindin 2; PCa: Prostate cancer; BCR: Biochemical recurrence; OR: Odds ratio; CI: Confidence interval; qRT-PCR: Quantitative real time reverse transcriptase polymerase chain reaction.

## Competing interests

The authors declare that they have no competing interests.

## Authors’ contributions

ZH, QC and XY conceived and designed the study, performed the experiments and wrote the paper. ZH and XY contributed to the writing and to the critical reading of the paper. ZH, QC, LL performed patient collection and clinical data interpretation. ZH and LL participated performed the statistical analysis. All authors read and approved the final manuscript.
